# Frequency of Efficient Circulating Follicular Helper T Cells Correlates with Dyslipidemia and WBC Count in Atherosclerosis

**DOI:** 10.29252/ibj.25.2.117

**Published:** 2021-01-13

**Authors:** Atefe Ghamar Talepoor, Shahdad Khosropanah, Mehrnoosh Doroudchi

**Affiliations:** 1 Department of Immunology, School of Medicine, Shiraz University of Medical Sciences, Shiraz, Iran;; 2 Department of Cardiology, School of Medicine, Shiraz University of Medical Sciences, Shiraz, Iran

**Keywords:** Atherosclerosis, Blood Platelets, Neutrophils, Dyslipidemias

## Abstract

**Background:**

The significance of cTfh cells and their subsets in atherosclerosis is not well understood. We measured the frequency of cTfh subsets in patients with different degrees of stenosis using flow-cytometry.

**Methods:**

Participants included high (≥50%; n = 12) and low (<50%; n = 12) stenosis groups, as well as healthy controls (n = 6).

**Results:**

The frequency of CCR7^lo^PD-1^hi^efficient-cTfh was significantly higher in patients with high stenosis compared to healthy controls (*p* = 0.003) and correlated with LDL (*p* = 0.043), cholesterol (*p* = 0.043), triglyceride (*p* = 0.019), neutrophil count (*p* = 0.032), platelet count (*p* = 0.024), NLR (*p* = 0.046), and PLR (*p* = 0.025) in high stenosis group. The frequency of CCR7^hi^PD-1^lo ^quiescent-cTfh was higher in healthy controls compared to the high-stenosis group (*p* = 0.001) and positively correlated with HDL (*p* = 0.046). The frequency of efficient-cTfh cells was correlated with platelet count (*p* = 0.043), NLR (*p* = 0.036), and PLR (*p* P = 0.035) in low-stenosis group, while that of quiescent-cTfh cells was negatively correlated with LDL (*p* = 0.034), cholesterol (*p* = 0.047), platelet count (*p* = 0.032), and PLR (*p* = 0.041).

**Conclusion:**

High percentages of cTfh and efficient-cTfh cells in patients with advanced atherosclerosis and their correlation with dyslipidemia and WBC counts suggests an ongoing cTfh subset deviation, towards efficient phenotype in the milieu of inflammation and altered lipid profile. Efficient cTfh cells have an effector phenotype and could in turn contribute to atherosclerosis progression.

## INTRODUCTION

Atherosclerosis is the most important source of cardiovascular morbidity and mortality worldwide^[^^1^^]^. As a chronic immune inflammatory disease, atherosclerosis is accompanied by the relocation of ox-LDL across the vasculature and activation of ECs^[^^2^^]^. This process is followed by innate and adaptive immune cells recruitment, foam cells formation, smooth muscle cells proliferation, plaques development, and eventually plaque rupture^[^^3^^]^. Nearly all the cellular elements in the blood, including WBC subtypes, monocytes, neutrophils, lymphocytes, red blood cells, and platelets, are involved in the pathogenesis of atherosclerosis^[^^3^^]^. Accordingly, the granule proteins of neutrophils lead to reactive oxygen species formation, endothelial dysfunction and vascular wall degeneration^[^^4^^]^. The innate immune cells of myeloid origin respond to modified lipoproteins and produce the inflammatory cytokines and chemokines^[^^4^^]^. Platelet granular substance also affects the activation of smooth muscle cells, ECs, and macrophages and cause atherosclerotic lesion development^[^^1^^]^. Besides, adaptive immune responses, orchestrated by T cells, may enhance inflammation^[^^5^^]^. The major antigens involved in T cells activation consist of epitopes of LDL and its core protein apolipoprotein B^[^^6^^]^, heat-shock protein 60/65 (HSP60/65)^[^^7^^]^, and peptides from pathogens such as HIV and cytomegalovirus^[^^6^^]^. Dendritic cells lead to the maturation and polarization of naive T cells through the presentation of antigenic peptides in the context of the major histocompatibility complex molecules, along with the secretion of chemokines and cytokines^[^^8^^]^. At the later stages of atherosclerosis, accumulation and interaction of immune cells with tissue-resident stromal cells and neovascularization lead to TLO formation^[^^9^^]^. Chronic inflammation fosters TLO neogenesis, and TLOs contribute to leukocyte recruitment and persistence in the atherosclerosis plaques where eventually affect the progression of the disease. CD4^+^ Th cells comprise a major population in human atherosclerotic plaques that can differentiate into diverse subtypes, including T helpers (Th1, Th2, and Th17), and Treg cells^[^^10^^]^. The most pathologic functions in atherosclerosis is played by Th1 and Th17 cells due to the production of inflammatory cytokines, as well as the stimulation of ECs and macrophages^[^^11^^]^. On the contrary, Treg and Th2 cells secrete anti-inflammatory cytokines and inhibit pathogenic T cells responses, thereby exerting a protective role in atherosclerosis^[^^6^^]^.

A distinct subset of peripheral blood CD4^+^ Th cells, cTfh cells, with exclusive functions, such as inducing B cell differentiation and antibody response, has recently been discovered^[^^12^^]^. cTfh cells are characterized as CD4^+^CXCR5^+^CD45RA^-^ cells that, unlike the germinal center Tfh cells, do not express BCL-6 transcription factor^[^^13^^]^. cTfh cells can be classified as efficient and quiescent by the combinations of the molecules viz CCR7, PD-1, and ICOS. PD-1^++ ^ICOS^+^CCR7^lo^cTfh cells are defined as efficient and PD-1^-^ICOS^-^CCR7^hi/int^ as quiescent cTfh^[^^14^^]^. The efficient cTfh cells produce IL-21, provide help for B cell and increase several inflammatory and autoimmune diseases^[^^14^^]^, whereas quiescent cTfh cells are known as non-efficient helper cells, which is accompanied by reduced antibody responses^[^^14^^]^.

Alternation in cTfh cell frequencies and functions has been reported in different autoimmune and inflammatory diseases, including systemic lupus erythematosus^[^^15^^]^, rheumatoid arthritis^[^^16^^]^, multiple sclerosis^[^^17^^]^, myasthenia gravis^[^^18^^]^, antineutrophil cytoplasmic antibody-associated vasculitis^[^^19^^]^, autoimmune thyroiditis^[^^20^^]^, chronic active hepatitis^[^^21^^]^, and CAD^[^^22^^]^. Therefore, imbalance in cTfh cell frequencies and phenotypes can participate in the pathogenesis of different diseases. However, the frequency of cTfh cells during the progression of atherosclerosis is still uncertain, and their phenotype and function in this disease remain to be delineated. 

In the current study, we aimed to investigate the frequency and phenotype of cTfh cells in patients with varied degrees of stenosis. In addition, we analyzed the association of cTfh cell subpopulations with clinicopathological manifestations in patients.

## MATERIALS AND METHODS

**Subjects**

In total, non-smoker, non-diabetic individuals with stenosis ≥50% (n= 12; mean age ± SD =58.41 ± 4.62 years) and stenosis <50% (n= 12, mean age ± SD = 50.83 ± 4.40 years) in coronary arteries were included in two case groups. Besides, six non-smoker, non-diabetic healthy individuals (three females and three males, mean age ± SD = 48 ± 3.03 years) were chosen as the control group. All subjects with stenosis were selected from individuals referred to hospitals affiliated to Shiraz University of Medical Sciences for diagnostic angiography. Exclusion criteria were: evidence or history of smoking, autoimmune diseases, diabetes, malignancy, inflammatory or infectious diseases in the last three months. Collecting demographic characteristics and clinical and laboratory data were carried out during admission. 

**PBMC isolation**

Fresh heparinized blood (30 ml) was obtained from each participant, and PBMCs were isolated by density gradient centrifugation using Ficoll-Paque Plus (GE Healthcare Europe, GmbH, Germany). Then the isolated cells were resuspended to 1 × 10^6^ per mL in RPMI-1640 culture medium (Shellmax, Iran) containing 10% fetal bovine serum and incubated at 37 °C overnight.

**Flow cytometry analysis**

Surface immunostaining of PBMCs was performed at 4°C for 20 minutes with the following human conjugated monoclonal antibodies: mouse anti-human CD3-Alexa Fluor 700, mouse anti-human CD4PerCP, mouse antihuman CXCR5-FITC, mouse anti-human CD45RA-PE/Cy7, mouse anti-human PD1-PE, and mouse anti-human CCR7-APC. We did not use isotype controls but utilized the single stained tubes as the basis of gating. All antibodies were purchased from BioLegend (San Diego, CA, USA). After washing twice with PBS, the samples were analyzed using FACS Aria II (BD Sciences, San Jose, USA); and the analysis of results was performed using FlowJo software (v7.6.2).

**Statistical analysis**

Kolmogorov-Smirnov test was carried out to assess the data normality. For frequency comparison of cTfh, efficient and quiescent cells among the groups, we used Kruskal-Wallis test. The correlation between variables was calculated using Spearman’s rank correlation test. The data were expressed as the mean ± SD and analyzed with SPSS version 18. A two-sided *p* value of <0.05 was considered statistically significant.

**Ethical statement**

The above-mentioned sampling protocols were approved by the Ethics Committee of Shiraz University of Medical Sciences, Shiraz, Iran (ethical code: IR.SUMS.REC.1397.1115). All study subjects provided written informed consent.

## RESULTS

**Demographic, clinical, and laboratory parameters**

Table 1 summarizes the demographic, clinical and laboratory characteristics of cases and controls. The serum level of TG (*p* = 0.0013) and LDL (*p* = 0.0014) as well as WBC (*p* = 0.0001), neutrophil count (*p* = 0.0001), platelet count (*p* = 0.0001), PLR (*p* = 0.0001), and NLR (*p* = 0.0001) were significantly higher in patients with stenosis ≥50% compared to other groups. Conversely, the serum level of HDL (*p* = 0.0116) and lymphocyte count (*p* = 0.0002) were higher in healthy controls compared to low and high stenosis groups, respectively. Additionally, there were no significant differences in BMI (*p* = 0.4543), systolic and diastolic blood pressure (*p* = 0.2035 and *p* = 0.1706, respectively), and serum level of cholesterol (*p* = 0.1) between patients and controls.

**Frequency of cTfh cells in PBMCs**

**Fig. 1:**
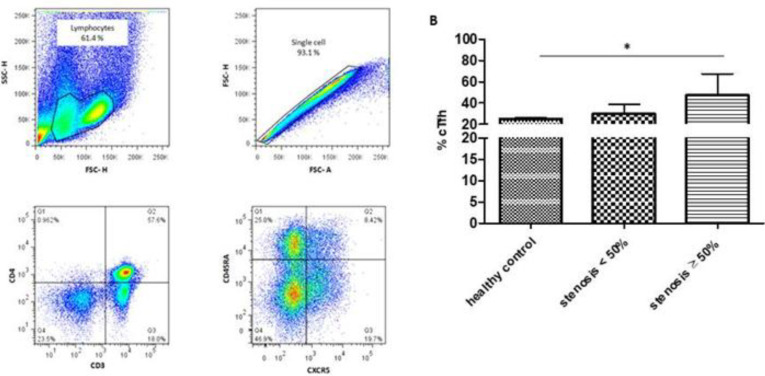
The frequency of cTfh cells was elevated in the blood of patients with stenosis ≥50% compared to the two other groups. (A) Gating strategy to detect CD4^+^CXCR5^+^CD45RA^-^cTfh in patients and controls. (B) The percentage of CD4^+^CXCR5^+^CD45RA^-^T cells within CD4+ T population in patients with stenosis ≥50% (n = 12), individuals with stenosis <50% (n = 12), and healthy controls (n = 6), Kruskal-Wallis test. ^*^p < 0.05

The frequency of cTfh cells was examined in the peripheral blood of all the groups. The CD4^+^CXCR5^+ ^CD45RA^-^Tfh cells in circulating PBMCs were identified by sequential surface marker gating as shown in Figure 1A. In addition, the percentage of cTfh cells significantly increased in patients with stenosis ≥50% compared to healthy controls (*p* = 0.002; Fig. 1B).

**Frequency of efficient cTfh cells in patients with atherosclerosis**

**Fig. 2:**
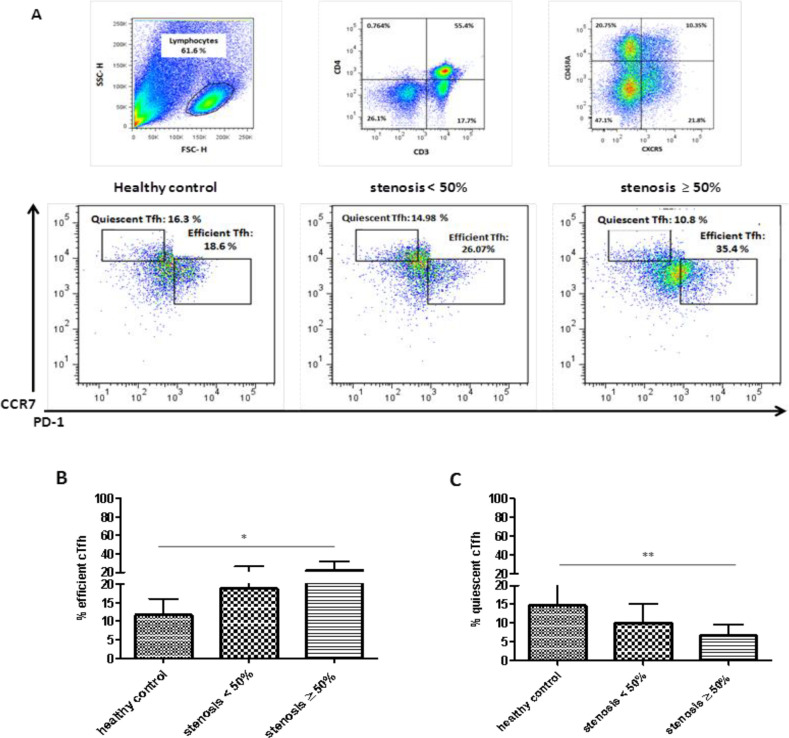
Comparison of efficient and quiescent cTfh subsets between the three groups.(A) cTfh subsets defined based on the expression of CCR7 and PD-1 on cTfh cells (i.e. CD4^+^CXCR5^+^CD45RA^-^CCR7^lo^PD-1^hi^ as efficient cTfh) and CD4^+^CXCR5^+^CD45RA^-^CCR7^hi^PD-1^lo ^defined as quiescent cTfh. Proportion of (B) efficient and (C) quiescent cTfh cells in individuals with stenosis ≥50% (n = 12), individuals with stenosis <50% (n= 12), and healthy controls (n = 6). Kruskal-Wallis test. *p < 0.05; ^**^p ≤ 0.01

In all studied groups, the expression of CCR7 and PD-1 molecules on the surface of cTfh cells was investigated (Fig. 2A). The frequency of CD4^+^CXCR5^+^CD45RA^- ^CCR7^lo^PD-1^hi^ efficient cTfh cells was significantly higher in patients with stenosis ≥50% than in the healthy controls (*p* = 0.003; Fig. 2B). In contrast, the frequency of the CCR7^hi^PD-1^lo^ CXCR5^+^CD45RA^-^CD4^+ ^quiescent cTfh cells was higher in the healthy controls compared to individuals with stenosis ≥50% (*p* = 0.001; Fig. 2C).

**Table 1:** The demographic, clinical and laboratory characteristics of the study participants. Data are shown as mean ± SD or cases number

**Parameters**	**Healthy controls** **(n = 6)**	**Stenosis <50%** **(n = 12)**	**Stenosis ≥50%** **(n = 12)**	***p*** ** value**
**Demography data**				
Gender (female/male)	3/3	6/6	6/6	
BMI (kg/m^2^)	24.6 ± 0.79	26.08 ± 3.53	25.09 ± 3.72	0.4543
				
**Clinical data**				
Systolic blood pressure (mmHg)	116.6 ± 5.1	129.5 ± 16.08	123.4 ± 21.4	0.2035
Diastolic blood pressure (mmHg)	75.8 ± 4.9	82.08 ± 14.09	72.5 ± 13.34	0.1706
				
**Laboratory data**				
TG (mg/dL)	113.1 ± 12.3	174.7 ± 39.3	219.5 ± 94.05	**0.0013**
Cholesterol (mg/dL)	160.0 ± 21.7	204.2 ± 57.8	193.5 ± 34.1	0.1039
LDL (mg/dL)	77.1 ± 24.07	121.8 ± 19.06	124.7 ± 20.5	**0.0014**
HDL (mg/dL)	55.0 ± 12.6	35.7 ± 5.9	39.3 ± 9.1	**0.0116**
WBC (10^3^/ μL)	7.9 ± 1.2	9.3 ± 0.45	10.8 ± 0.42	**0.0001**
Neutrophil (10^3^/μL)	4.9 ± 0.67	6.3 ± 0.41	8.5 ± 0.26	**0.0001**
Platelet (10^3^/micl)	152 ± 29.68	328.9 ± 59.2	495.08 ± 49.6	**0.0001**
Lymphocytes (10^3^/μL)	2.7 ± 0.43	2.6 ± 0.3	1.8 ± 0.21	**0.0002**
NLR	1.89 ± 0.1	2.38 ± 0.39	4.5 ± 0.47	**0.0001**
PLR	60.7 ± 20.89	121.5 ± 26.1	264.5 ± 34.09	**0.0001**

N, number; mmHg: millimeter of mercury

**Correlation of cTfh cells with clinical and laboratory parameters**

**Fig. 3:**
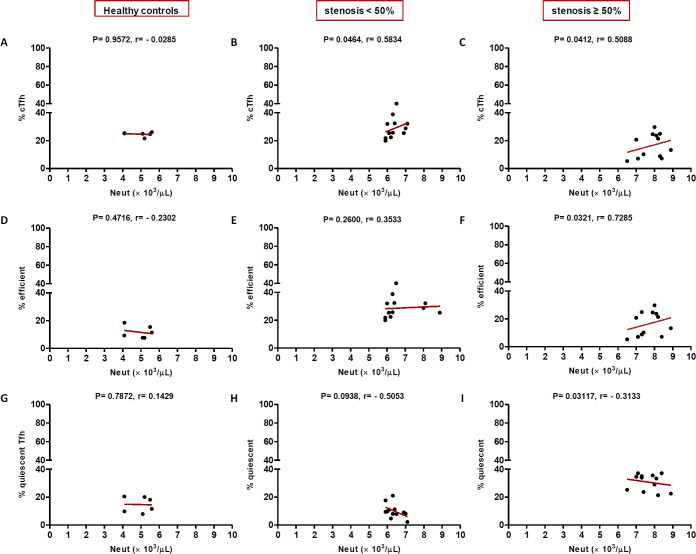
Correlation analysis. Correlation of the percentage of cTfh (A and C), efficient (D and F) and quiescent (G and I) cells with neutrophils count in all the three groups (Spearman’s rank correlation)

**Fig. 4:**
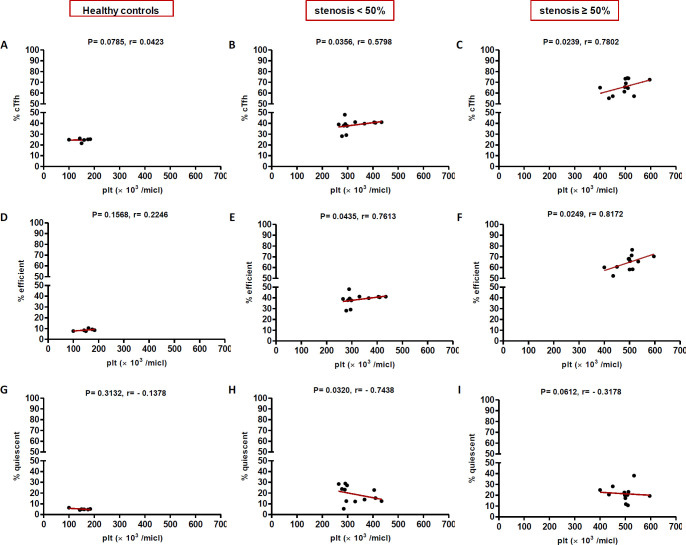
Correlation analysis. Correlation of the percentage of cTfh (A and C), efficient (D and F) and quiescent (G and I) cells with platelets count in all the three groups (Spearman’s rank correlation)

**Fig. 5:**
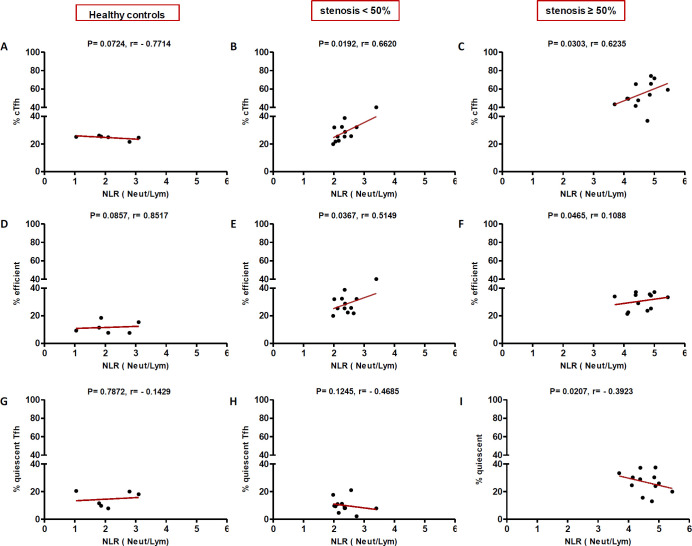
Correlation analysis. Correlation of the percentage of cTfh (A and C), efficient (D and F) and quiescent (G and I) cells with NLR in all the three groups (Spearman’s rank correlation)

**Fig. 6:**
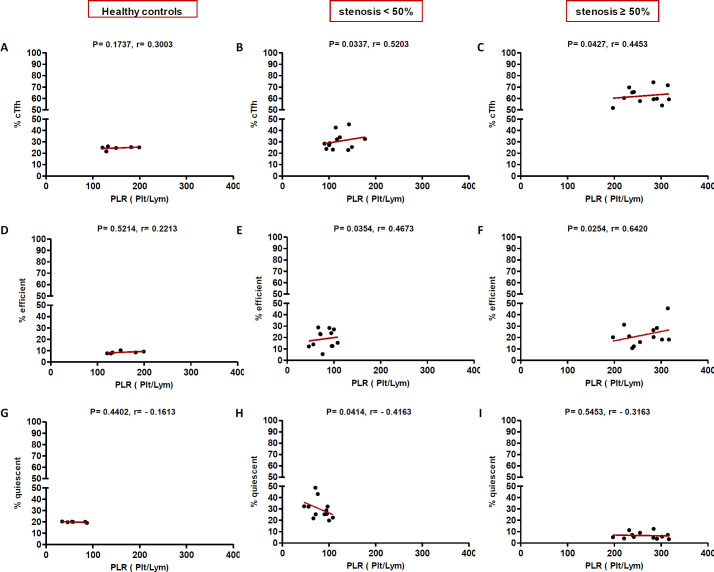
Correlation analysis. Correlation of the percentage of cTfh (A and C), efficient (D and F) and quiescent (G and I) cells with PLR in all the three groups (Spearman’s rank correlation)

**Fig. 7:**
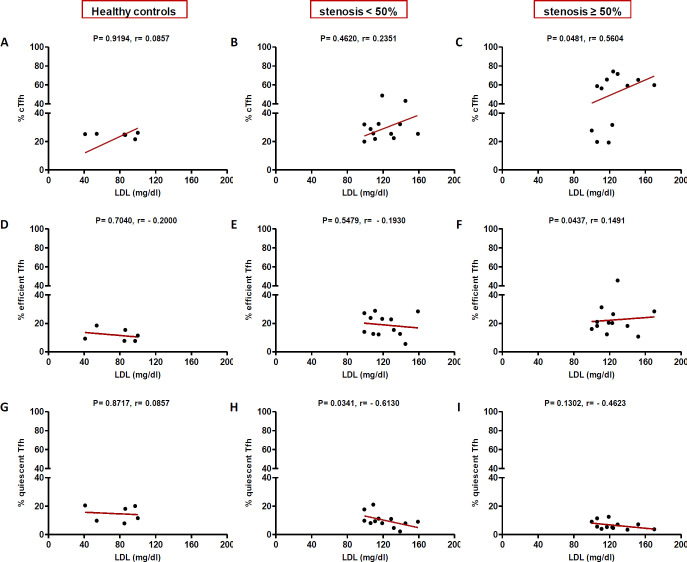
Correlation of the percentage of cTfh (A and C), efficient (D and F) and quiescent (G and I) cells with LDL in all the three groups (Spearman’s rank correlation)

**Fig. 8:**
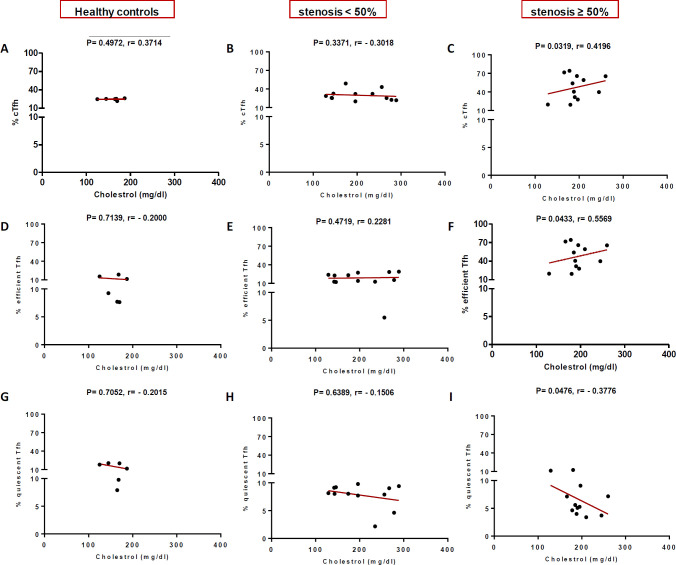
Correlation of the percentage of cTfh (A and C), efficient (D and F) and quiescent (G and I) cells with Cholesterol in all the three groups (Spearman’s rank correlation)

**Fig. 9:**
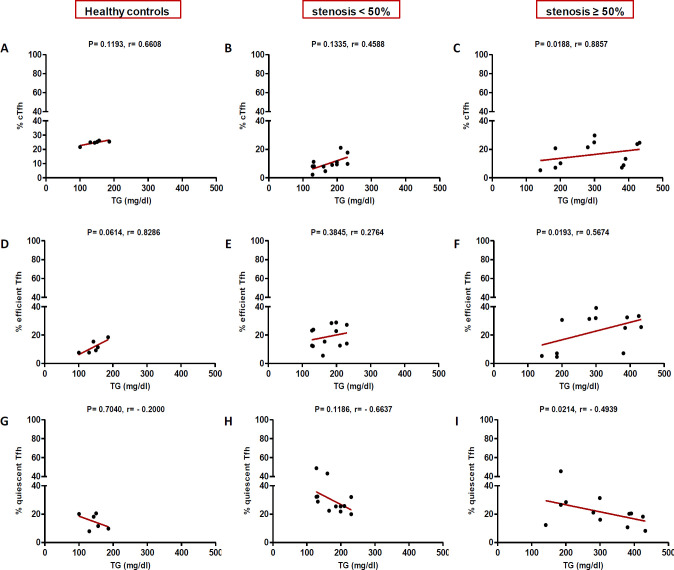
Correlation of the percentage of cTfh (A and C), efficient (D and F) and quiescent (G and I) cells with TG in all the three groups (Spearman’s rank correlation)

**Fig. 10:**
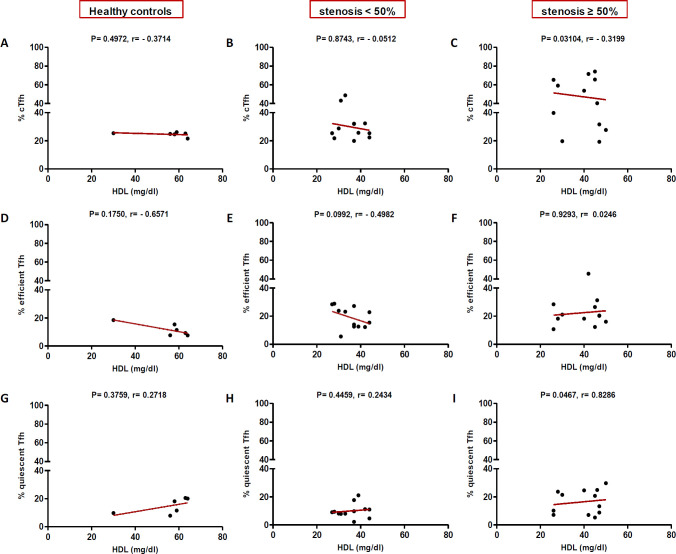
Correlation of the percentage of cTfh (A and C), efficient (D and F) and quiescent (G and I) cells with HDL in all the three groups (Spearman’s rank correlation)

**Fig. 11:**
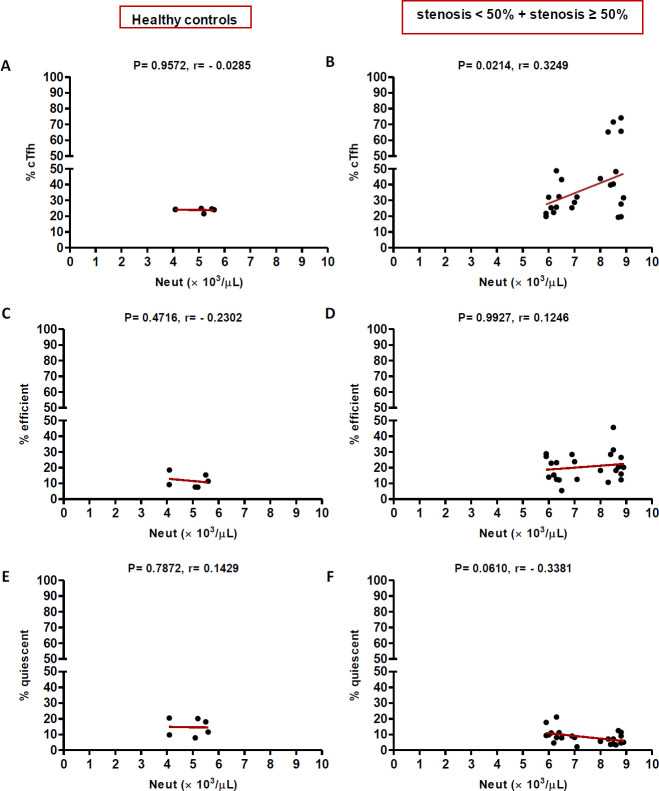
Correlation of the percentage of cTfh (A and B), efficient (C and D) and quiescent (E and F) cells with neutrophil count in healthy controls versus stenosis groups (Spearman’s rank correlation)

**Fig. 12:**
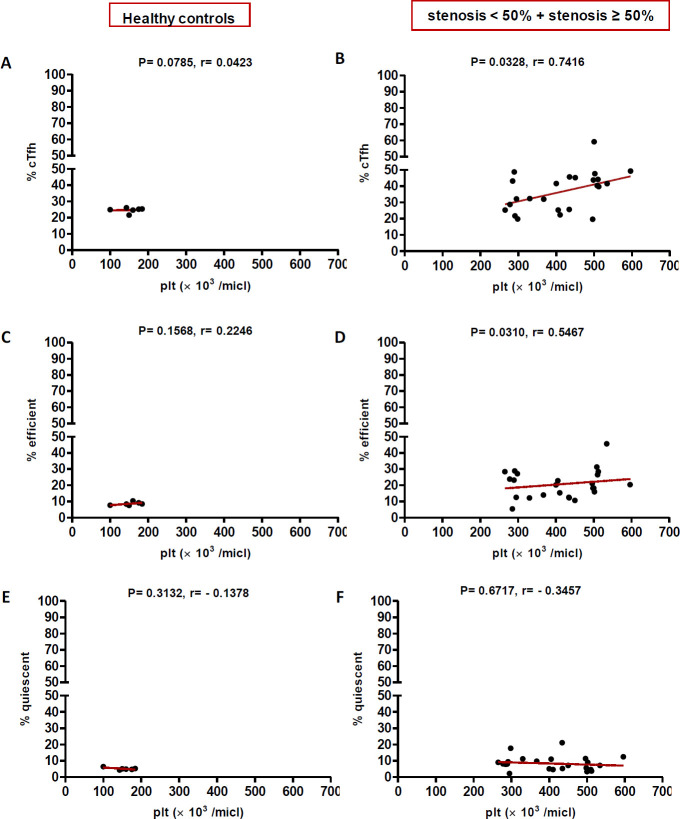
Correlation of the percentage of cTfh (A and B), efficient (C and D) and quiescent (E and F) cells with platelet count in healthy controls versus stenosis groups (Spearman’s rank correlation)

**Fig. 13:**
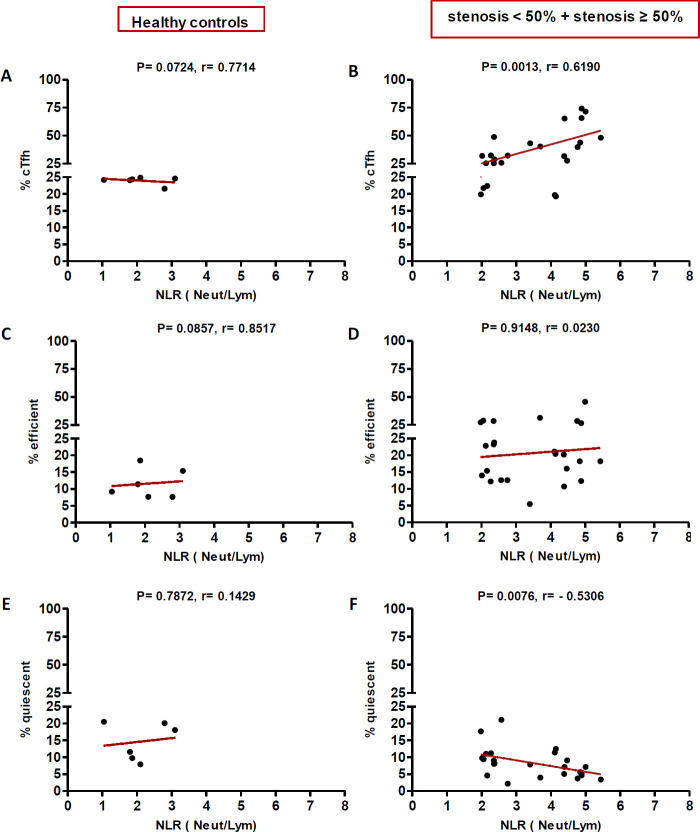
Correlation of the percentage of cTfh (A and B), efficient (C and D) and quiescent (E and F) cells with NLR in healthy controls versus stenosis groups (Spearman’s rank correlation)

**Fig. 14:**
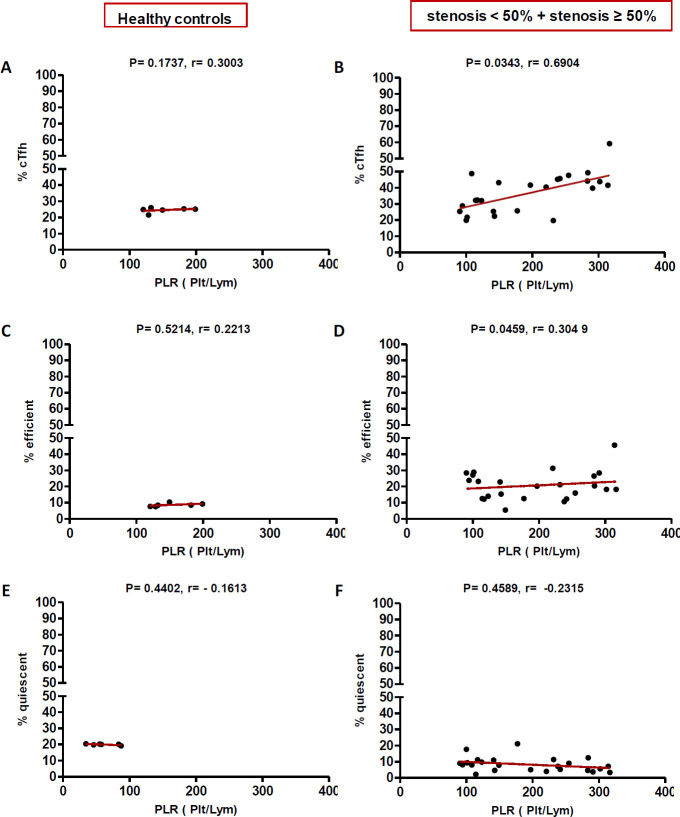
Correlation of the percentage of cTfh (A and B), efficient (C and D) and quiescent (E and F) cells with PLR in healthy controls versus stenosis groups, (Spearman’s rank correlation)

In high (≥50%) stenosis group, the percentage of cTfh and efficient cTfh cells were positively correlated with LDL (*p* = 0.048; r = 0.5604; Fig. 7C and *p* = 0.043; r = 0.1491; Fig. 7F, respectively) and TG (*p* = 0.018; r = 0.8857; Fig. 9C and *p* = 0.019; r = 0.5674; Fig. 9F, respectively). However, the association of cTfh cell percentage with HDL (*p* = 0.031; r = -0.3199; Fig. 10C) and of quiescent cTfh cells with TG (*p* = 0.021; r = -0.4939; Fig. 9I) was negative. In this group, the correlation of the frequency of cTfh and efficient cTfh cells with cholesterol (*p* = 0.031; r = 0.4196; Fig. 8C and *p* = 0.043; r = 0.5569; Fig. 8F, respectively) and that of quiescent cTfh cells with HDL (*p* = 0.046; r = 0.8286; Fig. 10I) were positive. Furthermore, the correlation of neutrophil (*p* = 0.031; r = -0.3133; Fig. 3I) and platelet (*p* = 0.032; r = -0.7438; Fig. 4H) counts and of NLR (*p* = 0.02; r = -0.3923; Fig. 5I) and PLR (*p* = 0.041; r = -0.4163; Fig. 6H) with quiescent cTfh cells were negative. In low stenosis group, the frequency of quiescent cTfh cells was negatively associated with LDL (*p* = 0.034; r = -0.6130; Fig. 7H) and cholesterol (*p* = 0.047; r = -0.3776; Fig. 8I). In high and low stenosis groups, as a whole, the percentage of cTfh cells was positively correlated with NLR (*p* = 0.07; r = 0.7714; Fig. 13A vs. *p* = 0.001; r = 0.6190; Fig. 13B) and PLR (*p* = 0.17; r = 0.3003; Fig. 14A vs. *p* = 0.034; r = 0.6904; Fig. 14B). Besides, in both low and high stenosis groups, there was a positive correlation of the cTfh frequency with NLR (*p* = 0.019; r = 0.6620; Fig. 5B and *p* = 0.03; r = 0.6235; Fig. 5C, respectively), PLR (*p* = 0.033; r = 0.5203; Fig. 6B and *p* = 0.042; r = 0.4453; Fig. 6C, respectively), neutrophil (*p* = 0.95; r = -0.0285; Fig. 11A vs. *p* = 0.021; r = 0.3249; Fig. 11B), and platelet counts (*p* = 0.07; r = 0.0432; Fig. 12A vs. *p* = 0.032; r = 0.7416; Fig. 12B), and platelet count (*p* = 0.035; r = 0.5798; Fig. 4B and *p* = 0.023; r = 0.7802; Fig. 4C, respectively). In contrast, the percentage of quiescent cTfh cells was negatively correlated with NLR (*p* = 0.007; r = -0.5306; Fig. 13F vs. *p* = 0.78; r = 0.1429; Fig. 13E). In both low and high stenosis groups, there was a positive correlation between the frequency of efficient cTfh cells with PLR (*p* = 0.035; r = 0.4673; Fig. 6E and *p* = 0.025; r = 0.6420; Fig. 6F, respectively). Also, increase in the frequency of efficient cTfh cells was positively correlated with neutrophil count (*p* = 0.032; r = 0.7285; Fig. 3F) and with PLR (*p *= 0.04; r = 0.3049; Fig. 14D vs. *p* = 0.52; r = 0.2213; Fig. 14C), platelet counts (*p* = .031; r = 0.5467; Fig. 12D vs. *p* = 0.15; r = 0.2246; Fig. 12C), and NLR (*p* = 0.036; r = 0.5149; Fig. 5E and *p* = 0.046; r = 0.7088; Fig. 5F, respectively). In stenosis groups and healthy controls, the percentage of cTfh cells was not associated with BMI, SBP and DBP, and no significant correlation was observed. In both early and late atherosclerosis, a positive correlation was observed between the frequency of efficient cTfh cells and platelet count (*p* = 0.043; r = 0.7613; Fig. 4E and *p* = 0.024; r = 0.8172; Fig. 4F, respectively).

## DISCUSSION

We first investigated the percentage of cTfh cells in patients with atherosclerosis. Our result demonestrated that the frequency of cTfh cells was higher in patients with high (≥50%) stenosis than in healthy controls. Our findings support the results of the findings by Ding *et al.*’s^[^^22^^] ^findings on the increased frequency of CD4^+^CXCR5^+^PD-1^+^CCR7^-^T cells in patients with CAD. Also, previous studies in other Type 2 diabetes mellitus^[^^23^^]^, non-small cell lung cancer^[^^24^^]^, osteosarcoma^[^^25^^]^, antineutrophil cytoplasmic antibody-associated vasculitis^[^^19^^]^, psoriasis vulgaris^[^^26^^]^, and chronic lymphocytic leukemia^[^^27^^]^ showed similar results.

The investigation of the frequency of efficient and quiescent cTfh cells in atherosclerosis indicated an increase in the frequency of efficient (CD4^+^CXCR5^+ ^CD45RA^-^CCR7^lo^PD-1^hi^) cTfh cells in individuals with high stenosis, accompanied by a decrease in the quiescent (CD4^+^CXCR5^+^CD45RA^-^CCR7^hi^PD-1^lo^) cTfh cells. A recent study showed that in patients with myositis, IL-21 expressing CCR7^lo ^PD-1^hi^ efficient cTfh cells significantly increased, and the frequencies of these cells had a correlation with the disease progression^[^^28^^]^. Moreover, the percentage of ICOS^hi^PD-1^hi^ efficient cells was higher in patients with Kawasaki syndrome^[^^29^^]^. The ICOS^+^PD-1^+^ cTfh cells and IL-21 producing CD4^+^CXCR5^+^PD-1^+^ T cells were also shown to be significantly higher in patients with Sjögren syndrome^[^^30^^]^. In addition, cTfh cells in CAD patients are indicated to be enriched with PD-1^hi ^CCR7^lo^ subset, which secreted higher IFN-γ, IL-17A, and IL-21 upon stimulation^[^^22^^]^. Therefore, it is logical to assume that cTfh cells might contribute to pathogenesis of atherosclerosis through the enrichment of PD-1^+^CCR7^−^ efficient subset and inflammatory cytokines secretion. 

In our patients with high stenosis, the percentage of cTfh and efficient cTfh cells were positively correlated and that of quiescent cTfh cells were negatively correlated with cholesterol, LDL, or TG (Figs. 7-10). In general, the increase in the efficient cTfh cells was accompanied by an elevation in cholesterol and LDL later in the disease progression (i.e. in patients with high stenosis). The decrease in the quiescent cTfh cells, however, was accompanied by enhancement in the LDL, TG, and HDL earlier in the disease (i.e. in individuals with low stenosis). While the association of LDL and dyslipidemia with atherosclerosis and its progression is well established^[^^31^^,^^32^^]^, its relationship with T-cell subsets and their deviations is just recently being unfolded. In this regard, Li *et al.*^[^^33^^]^ investigated the effects of ox-LDL on Th17/Treg cell apoptosis and proliferation in patients with atherosclerotic cerebral infarction. They found that the high concentration of ox-LDL lead to decrease in the frequency and function of Treg cells and increase in the frequency of Th17 cells. Emerging evidence has indicated that cholesterol and fatty acid biosynthesis programs are upregulated during Th17 differentiation^[^^34^^]^. A recent study has also represented the effect of hyperlipidemia on the Tfh cells frequency and function in atherosclerosis-associated systemic lupus erythematosus in both mice and humans^[^^35^^]^. It has been shown that the increased level of IL-6, IFN-β, and IL-27 in the sera of ApoE-deficient atherogenic mice and IL-27 is sufficient to induce cTfh cells and germinal center reactions in these mice. Of note, the higher level of IL-27 in patients with hypercholesterolemia is ascribed to cTfh cells function and increased immunoglobulin G in their circulation^[^^35^^]^. In addition, the expression and signaling of IL-2R, a potent inhibitor of Tfh differentiation, reduce concomitant with a raise in the intracellular cholesterol level. Consequently, cholesterol-mediated attenuation of IL-2 signaling, along with the increased IL-6R and Bcl6 expression, initiate Tfh differentiation in the context of atherosclerosis^[^^36^^]^.

In contrast to atherogenic lipids, HDL inhibits cytokine-induced expression of adhesion molecules on ECs and stimulates cholesterol reverse transport to the liver, thereby protecting against atherosclerosis^[^^37^^]^. ApoAI is the main protein component of HDL without which plasma HDL levels would reduce^[^^38^^]^. ApoAI can decrease the maturation of dendritic cells and T cell responses during inflammation^[^^39^^]^. During atherosclerosis progression, Treg cells switch their phenotype into pro-atherogenic Tfh cells, which are responsible for the formation of tertiary lymphoid structures in the aorta and B cell-mediated antibody production^[^^40^^]^. ApoAI inhibits Treg to Tfh cell conversion during atherosclerosis through the regulation of cholesterol levels and IL-2 receptor expression in Treg cells^[^^41^^]^. In a previous study, it has been shown that ApoAI administration reduces cholesterol-mediated effector T cell expansion and increases Treg cells in Ldlr^−/−^ApoAI^−/−^ mice^[^^42^^]^. Therefore, dyslipidemia is involved in atherosclerosis progression not only through the regulation of antigen presentation, cytokine production, immune cell activation, proliferation and migration but also through the manipulation of T cell subset deviation^[^^43^^]^. In this regard, our study is the first to indicate alterations in cTfh subsets in atherosclerosis are correlated with dyslipidemia and the state of disease progression. Furthermore, positive correlations were found between the frequency of cTfh cells subsets and that of neutrophils and platelets, and their ratios indicated as NLR and PLR. In our study, both neutrophil and platelet counts positively correlated with cTfh percentage in low and high stenosis groups. The increase in the frequency of efficient cTfh cells positively correlated with neutrophil count later in the disease, while the positive correlation between platelet count and frequency of efficient cTfh cells observed in both the early and late atherosclerosis. Interestingly, the association of both neutrophil and platelet counts with quiescent cTfh cells were negative and followed the same pattern. The elevation in the frequency of efficient cTfh cells had a positive correlation with NLR and PLR in both early and late atherosclerosis. Different white blood cell subtypes, including neutrophils, lymphocytes, and platelets, are important players in the pathogenesis of atherosclerosis^[^^44^^]^. In addition, NLR and PLR are introduced as reliable inflammatory biomarkers, pointing to the balance between the innate and adaptive immune responses^[^^45^^]^. The ongoing inflammation during atherosclerosis leads to decreased lymphocyte counts and increased neutrophils and platelets proliferation^[^^46^^]^. Former studies have demonstrated that high NLR/PLR and increased neutrophil/platelet counts are associated with the severity and progression of cardiovascular diseases^[^^47^^-^^49^^]^. Therefore, our findings may indicate the potential transformation of quiescent Tfh cells to efficient Tfh cells, along with the inflammatory process orchestrated by neutrophils and platelets or other cells.

In conclusion, the high frequencies of cTfh and efficient cTfh cells in patients with high stenosis and their correlation with lipid profile and WBC counts, suggest that these cells, as effectors, are important in immune responses related to atherosclerosis. It would, therefore, be interesting to investigate the significance of cTfh cells and their functional subsets in the progression of stenosis and the prediction of disease outcome in patients with CAD in a large longitudinal cohort of patients.
